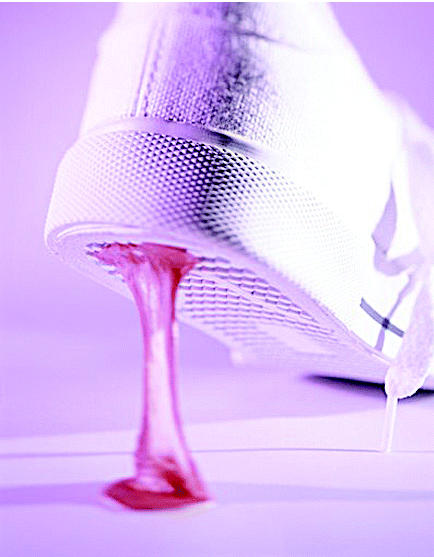# The Beat

**Published:** 2005-10

**Authors:** Erin E. Dooley

## Mercury’s Afterlife?

A report released by the New England Zero Mercury Campaign says that dental fillings in cremated corpses emit about 2.5 tons of mercury each year, with the amount expected to double by 2025. Dental amalgams are 50% mercury by weight. Although the use of mercury-containing amalgams is declining steadily, 34 tons of mercury are still used for dental purposes each year. The EPA has met with the American Dental Association to discuss ways to further reduce the use of mercury in fillings.

More people today die with some or all of their teeth in place. In addition, more people are choosing cremation. The April 2005 report is online at http://www.cleanwateraction.org/mercury/pdf/NEZMC_ReportCard_DentalMercury.pdf.

## Climate Change Hits the Road

Cities are the biggest consumers of electricity and therefore the primary generators of the greenhouse gases that cause global warming. Now the British Council, Great Britain’s international agency for promoting education and cultural relations, has launched its US$7-million two-year ZeroCarbonCity campaign to educate people in 100 cities across 60 countries about climate change. The campaign, begun in March 2005, teaches how decisions at all levels, from urban planning to personal choices people can make every day, can contribute to or help mitigate the effects of climate change. A traveling photographic exhibition is visiting all 100 cities as part of the campaign. Related debate transcripts and publications are available online at http://www.britishcouncil.org/zerocarboncity.htm?mtk=8.

## Personal Products Keep Organic Label

People who prefer to buy organic cosmetics, dietary supplements, and pet food can breathe a sigh of relief—in August 2005, the USDA ruled that the use of the “USDA Organic” label is permissible on those products. The ruling reverses an earlier decision that putting the green and white label on such items went beyond the original intent of the labeling program, implemented in 2002. Following the original ruling, the Dr. Bronner’s Magic Soaps organic body care company and the Organic Consumers Association filed a suit against the USDA, a move seen as the leading factor behind the reversal.

## Taiwan Touts Trash Sorting

Taiwan, with the acreage of Belgium but twice the population, has 200 landfills. In two years these will be full, leaving the island nation dependent on some 20 trash incinerators that emit pollutants such as dioxin. To help curb the flow of waste into the incinerators, new recycling laws have been enacted that fine residents almost US$200 for not sorting their trash properly.

The laws, now in force only in Taipei, will be implemented across Taiwan by January 2006 in an effort to cut the number of trash incinerators to five within 20 years. Over 90% of Taipei residents are reportedly complying with the new rules.

## Pandemic Prevention

Officials from 192 countries agreed upon revised regulations for notifying the WHO of all major disease outbreaks and suspected bioterrorism events at a May 2005 meeting. Until now, only outbreaks of cholera, plague, and yellow fever had to be reported to the organization. The regulations, which come into effect in 2007, also require that the WHO assist member countries in responding to such threats and in fostering greater international cooperation in outbreak response. The health ministers and other officials who signed the regulations hope the new system will help contain outbreaks of infectious diseases such as SARS and influenza before they spread globally.

## Less Gummy Gum

There’s no doubt about it: chewed chewing gum is hard to clean up if it ends up anywhere except in a trashcan. In the United Kingdom alone, over US$260 million is spent each year by municipalities on gum cleanup, and the methods used include abrasive chemical cleaners, pressure washing, and scraping. Now the University of Manchester in England and the company Green Biologics are developing a biologically based cleaner, TP-GUM™, that is cost-effective and nontoxic. The new product, which uses enzymes to break down the chemical structure of chewing gum at low temperatures and pressure, is easier to use and less damaging to surfaces than conventional gum cleanup methods.

## Figures and Tables

**Figure:**
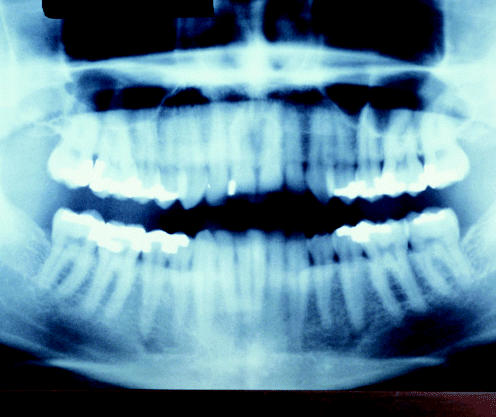


**Figure:**
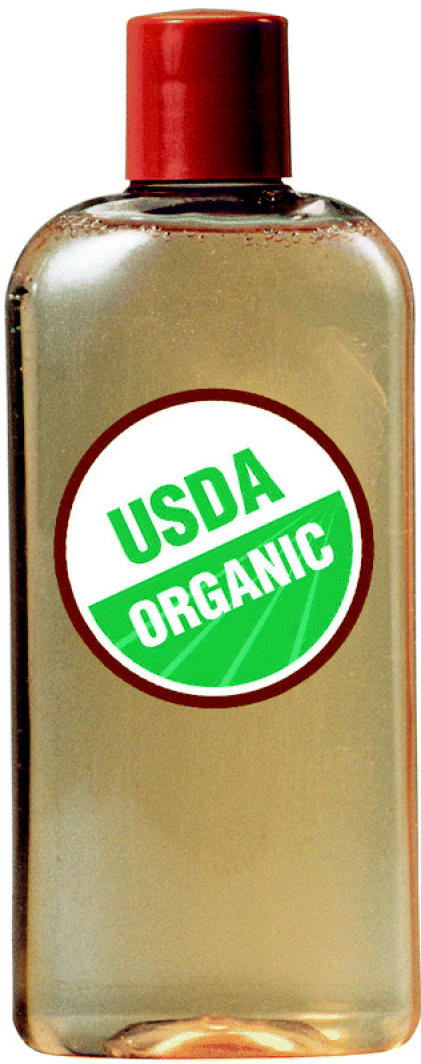


**Figure:**
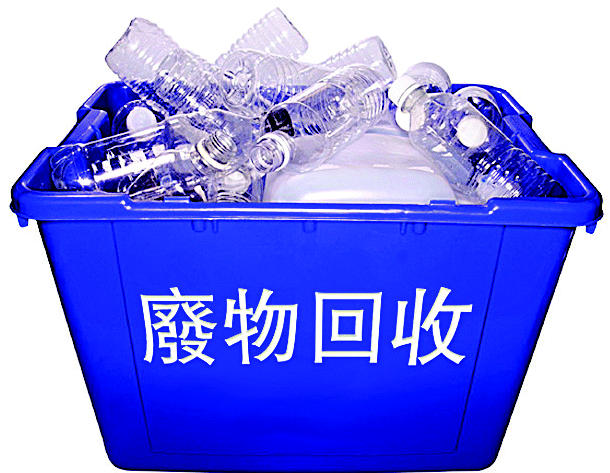


**Figure:**